# Building on Capacity Established through US Centers for Disease Control and Prevention Global Health Programs to Respond to COVID-19, Cameroon

**DOI:** 10.3201/eid2813.221193

**Published:** 2022-12

**Authors:** Emily Kainne Dokubo, Judith D. Shang, Adama N’Dir, Clement B. Ndongmo, Gordon Okpu, Yasmine Moussa Fadil, Laura Eno Takang, Carrine Angumua, Esther Lyonga, Magdalene Mayer, Tabiayuk Ayukotabe, Tse K. Nkwoh, Judith Hedje, Georges A. Etoundi, Richard L. Njock, for the Centers for Disease Control and Prevention Cameroon Team

**Affiliations:** US Centers for Disease Control and Prevention, Atlanta, Georgia, USA (E.K. Dokubo, A. N’Dir, C.B. Ndongmo, J. Hedje);; US Centers for Disease Control and Prevention, Yaoundé, Cameroon (J.D. Shang, G. Okpu, Y.M. Fadil, L.E. Takang, C. Angumua, E. Lyonga, M. Mayer, T. Ayukotabe, T.K. Nkwoh);; Ministry of Public Health, Yaoundé (G.A. Etoundi, R.L. Njock)

**Keywords:** COVID-19, coronavirus disease, severe acute respiratory syndrome coronavirus 2, SARS-CoV-2, respiratory infections, global health, outbreak response, HIV, tuberculosis, zoonoses, Cameroon

## Abstract

The COVID-19 pandemic has highlighted the need for resilient health systems with the capacity to effectively detect and respond to disease outbreaks and ensure continuity of health service delivery. The pandemic has disproportionately affected resource-limited settings with inadequate health capacity, resulting in disruptions in health service delivery and worsened outcomes for key health indicators. As part of the US government’s goal of ensuring health security, the US Centers for Disease Control and Prevention has used its scientific and technical expertise to build health capacity and address health threats globally. We describe how capacity developed through global health programs of the US Centers for Disease Control and Prevention in Cameroon was leveraged to respond to coronavirus disease and maintain health service delivery. The health system strengthening efforts in Cameroon can be applied in similar settings to ensure preparedness for future global public health threats and improve health outcomes.

The ongoing COVID-19 pandemic was declared a public health emergency of international concern by the World Health Organization (WHO) on January 30, 2020 (*1*). As of July 13, 2022, the pandemic had affected >232 countries and territories, resulting in >555.4 million cumulative COVID-19 cases and 6.3 million deaths globally (*2*). The effect of the pandemic has been far-reaching. It has caused major disruptions to essential health services in almost all countries globally, exacerbating gaps in health systems with weak infrastructures and undoing global health gains in nearly all major health areas (*3*).

Cameroon is a low to middle-income country in central Africa that has a limited domestic health expenditure of ≈4% of its gross domestic product and poor outcomes for key health indicators. Concurrent security and humanitarian crises have further affected the health system, including Boko Haram and ISIS-West Africa terrorist attacks in the Far North region (*4*); ongoing civil conflict and worsening violence in the Anglophone Northwest and Southwest regions (*5*); and large settlements of refugees in the Northern and East regions (*6*) from neighboring Central African Republic, Nigeria, and Chad. The country has been greatly affected by the COVID-19 pandemic and accounts for the highest number of COVID-19 cases and deaths in Central Africa (*7*). For much of the first year of the pandemic, the COVID-19 case count and case-fatality rate for Cameroon were among the highest in Africa, and as of March 16, 2022, two years since the start of the pandemic in Cameroon, there were 119,544 confirmed cases, including 1,927 deaths, and a case-fatality rate of 1.6% (*2*). Health personnel accounted for 4,419 confirmed cases and 61 deaths, reflecting the disproportionate effect of the pandemic on the health workforce.

The US Department of Health and Human Services and Centers for Disease Control and Prevention (CDC) began work in Cameroon during 1998 by establishing an HIV laboratory and research program. The presence of CDC in Cameroon evolved to an established country office in 2004, providing technical expertise and support to the Ministry of Health (MOH) to strengthen disease control efforts and develop sustainable public health capacity. In 2007, an agreement was signed between the US and Cameroon governments, establishing a partnership to prevent and control HIV/AIDS, avian influenza, and other infectious diseases. Consistent with the International Health Regulations (2005) that states WHO member states should develop, strengthen, and maintain their capacity to respond promptly and effectively to public health emergencies of international concern (*8*), Cameroon has focused on strengthening its capacity to respond to public health threats with support from the US government and other partners

CDC provides technical and financial assistance to the Cameroon MOH at the national and subnational levels and delivers clinical services in >300 health facilities across all 10 regions of the country through implementing partners. Health systems strengthening efforts include building epidemiology, surveillance, laboratory, research, and emergency management capacity and developing a fit-for-purpose workforce to ensure the sustainability of programs. As a key implementing agency of the President’s Emergency Plan for AIDS Relief (*9*), CDC has scaled-up HIV prevention, care, and treatment services for persons living with HIV and accelerated progress in controlling the HIV epidemic in Cameroon. Through the Global Health Security Agenda (*10*), CDC has strengthened the capacity of Cameroon to prevent, detect, and effectively respond to disease outbreaks. Implementation by CDC of the President’s Malaria Initiative (*11*) to reduce malaria-related illness and death, technical assistance for vaccine-preventable diseases, and support for other global health programs have contributed to health system strengthening in Cameroon.

## Leveraging Global Health Programs of CDC for COVID-19 Response

Building on the strong partnership between CDC and Cameroon MOH, Cameroon leveraged the capacity established through US government‒funded global health programs to prepare for and respond to the COVID-19 pandemic. With support from CDC, WHO, and other technical partners, the Cameroon MOH initiated outbreak preparedness planning in January 2020 when COVID-19 was designated as a public health emergency of international concern, developed a COVID-19 preparedness and response plan, and conducted trainings for health officials at national and subnational levels. In addition, Cameroon hosted in March 2020 a meeting of the Health Ministers of the Economic and Monetary Community of Central Africa to develop a joint plan for prevention, preparation, and response to COVID-19 in the Central Africa region.

After detection of the first COVID-19 case in Cameroon on March 6, 2020, the National Public Health Emergency Operations Center (PHEOC) was activated for the response. The PHEOC is a state-of-the-art facility constructed and established with support from the US Defense Threat Reduction Agency and CDC and handed over to the government of Cameroon in June 2019 to ensure coordination and management of health emergencies (*12*). Functioning of the PHEOC requires well-trained emergency management experts. To this end, CDC’s Public Health Emergency Management Fellowship in Atlanta builds the emergency management capacity of international health officials through specialized training, mentorship, and technical assistance (*13*). Eleven senior Cameroon health officials trained through the fellowship were essential to the stand-up of the PHEOC and lead different aspects of the COVID-19 response. CDC has also strengthened emergency management capacity at the subnational level by supporting the establishment of rapid response teams in the 10 regions of Cameroon, which have responded to multiple disease outbreaks and public health emergencies. After the activation of the National PHEOC, the emergency operations centers and rapid response teams in all regions of Cameroon were activated for the COVID-19 response.

**Figure 1:**
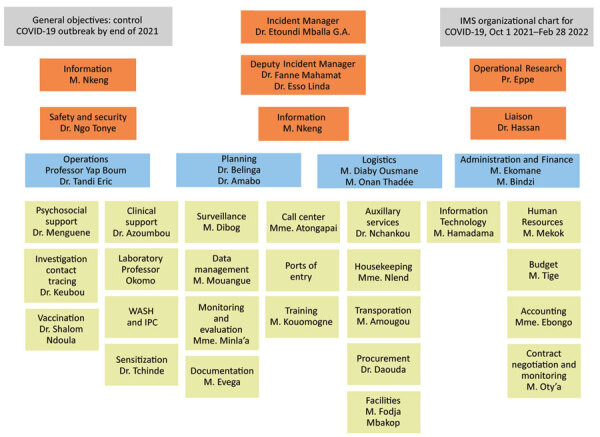
Cameroon Ministry of Health COVID-19 incident command structure and pillars supported by the Centers for Disease Control and Prevention. Source: Cameroon Ministry of Health/Public Health Emergency Operations Center. IMS, Information Management System; IPC, Integrated Phase Classification.

The CDC office in Cameroon organized a COVID-19 Response Team comprising staff who had previously supported the 2014–2016 West Africa Ebola response and other disease outbreaks, applying their outbreak response expertise to support COVID-19 response efforts. CDC developed a response plan aligned with the COVID-19 plan of the Cameroon MOH. CDC public health experts were integrated into the National Incident Management System (IMS), an established command structure to manage emergency responses (*14*), and provided technical leadership and expertise in conjunction with WHO for the response efforts of MOH. The CDC COVID-19 Response Team members provided expert technical support across all pillars of the National IMS, including surveillance, laboratory, case management, and infection prevention and control (Figure 1). Coordination of partners involved in the response increased efficiencies and helped to address duplication of efforts by multiple stakeholders.

**Figure 2:**
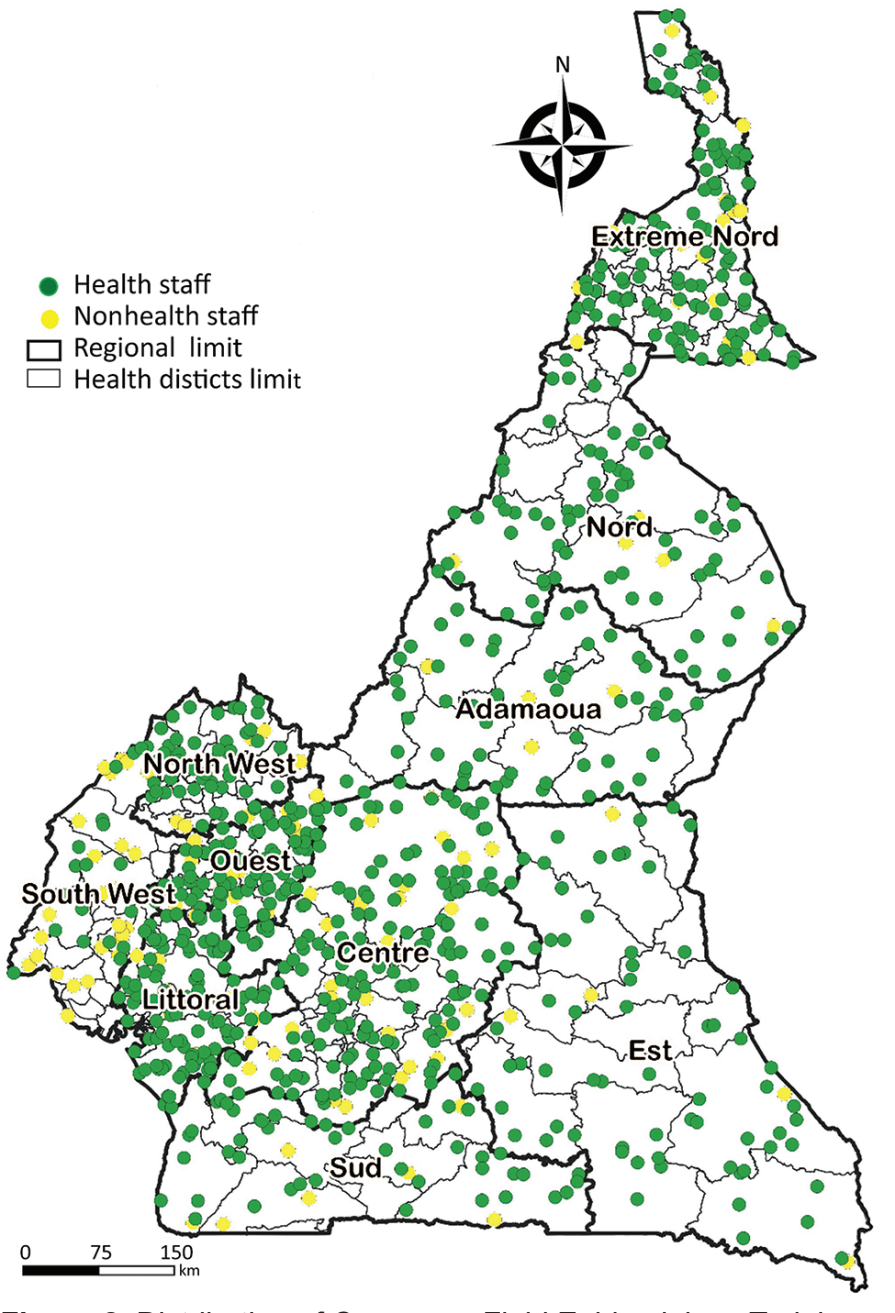
Distribution of Cameroon Field Epidemiology Training Program trainees and graduates by region, Cameroon, July 2022. Source: Cameroon Ministry of Health/Cameroon Field Epidemiology Training Program.

The CDC-established Field Epidemiology Training Program (*15*) developed a trained global public health workforce to collect, analyze, and interpret data for decision making, strengthening countries’ capacities to address public health challenges and meet the needs of their population. Established in 2010, Cameroon’s Field Epidemiology Training Program (CAFETP) trains health officials in Cameroon and neighboring countries in central Africa to strengthen the public health workforce in the region. More than 1,100 CAFETP graduates and trainees distributed across the country (Figure 2) supported the National and Regional IMS and were the ground force of the COVID-19 response of Cameroon, constituting rapid response teams and conducting disease surveillance, case investigations, and contact tracing. With oversight from the CDC Field Epidemiology Training Program Resident Advisor, CAFETP graduates, and trainees conducted active surveillance, monitored contacts of cases, and enabled early detection and management of COVID-19 cases. The CAFETP also trained health staff working in prisons on case investigation and mitigating transmission risk in congregate settings. Border health measures were put in place at different points of entry to reduce the risk for transmission from travelers to Cameroon. In collaboration with other technical partners, CDC supported the MOH in developing passenger screening protocols; training health officials to conduct screenings at the international airports, seaports, and land border crossings; implementing COVID-19 testing at points of entry; establishing isolation and quarantine measures for passengers upon arrival; and conducting supportive supervision of border health officials.

CDC helped to develop and strengthen the capacity of laboratories in Cameroon by supporting establishment and renovation of the National Public Health Laboratory in 2016, leading the development of the first National Laboratory Strategic Plan in 2018, and leading the implementation of the Strengthening Laboratory Improvement Process Toward Accreditation, a framework for evaluating the progress of laboratories toward international accreditation (*16*). As a result of CDC support, 5 laboratories have received ISO-15189 accreditation (https://anab.ansi.org), meeting international standards for quality management systems and competence for medical laboratories (*17*). Those 5 laboratories were the first internationally accredited laboratories in Cameroon and central Africa. Cameroon was among the first countries in central Africa that had COVID-19 diagnostic capacity. At the start of the pandemic, Centre Pasteur Cameroon was the only reference laboratory for COVID-19 testing, but testing capacity quickly became overwhelmed because of increased testing needs for samples from all regions of the country.

The challenges with testing called attention to the need for enhanced collaboration between the Cameroon MOH and stakeholders to manage human resources and ensure timely procurement and management of reagents and testing commodities. CDC decentralized the response by providing technical support for COVID-19 testing and development of a laboratory strategy in Cameroon and a decentralization plan to expand capacity from the national level to a network of 19 laboratories across the country. The National Public Health Laboratory coordinated the distribution of COVID-19 test kits and received CDC support to procure sample collection and transportation material, improve laboratory supply management, and establish a call center to ensure reporting of results from decentralized laboratories. CDC staff conducted supervisory visits to laboratories to provide technical support on workflow, strengthen biosafety measures, and validate COVID-19 testing algorithms. CDC supported the expansion of testing strategies, mobile testing units for sample collection, and delivering negative test results through text messages, which led to increased access to testing and reduced turnaround time for test results. As of March 16, 2022, PCR testing had been conducted on 608,118 samples and rapid antigen testing on 1,916,552 samples. CDC also supported genomic surveillance to detect new circulating variants of SARS-CoV-2.

The CDC COVID-19 response staff embedded in the National IMS pillar for case management (Figure 1) provided support to improve outcomes for confirmed cases. Efforts included supporting development of case management algorithms, standard operating procedures, and registers; the training of health workers on patient management; and conducting field supervision visits to COVID-19 isolation and treatment centers. Establishing a community of practice among case management physicians resulted in weekly sharing of data and best practices among COVID-19 isolation and treatment centers, leading to improved outcomes. CDC also participated in joint assessments of COVID-19 treatment centers with WHO and the MOH and developed a supportive supervision tool used to assess the functional capacity of treatment centers and provide recommendations to improve gaps. Infection prevention and control (IPC) measures were focused on preventing nosocomial transmission among patients and healthcare workers. CDC provided personal protective equipment and IPC supplies for health facilities and healthcare workers, supported IPC guideline development, conducted trainings for health workers on IPC practices, and participated in supportive supervision visits with WHO and the MOH.

Risk communication and community engagement are key components of outbreak response (*18*). CDC supported development and implementation of the national communication plan for COVID-19 in Cameroon, developed and disseminated risk communications tools, and established and supported call centers at national and regional levels. CDC also supported the intersectoral approach to achieving community ownership and engagement at all levels and provided technical support for public communication and press briefings on COVID-19 preparedness and response efforts in Cameroon. CDC COVID-19 response staff were integrated into the National IMS communication pillar, and culturally appropriate health messages to counter COVID-19 misinformation were developed for print, broadcast, and social media. CDC also supported revisions of the communication strategic plan and tools to meet the changing communications needs of the outbreak and sensitize the population to COVID-19 vaccines.

**Figure 3:**
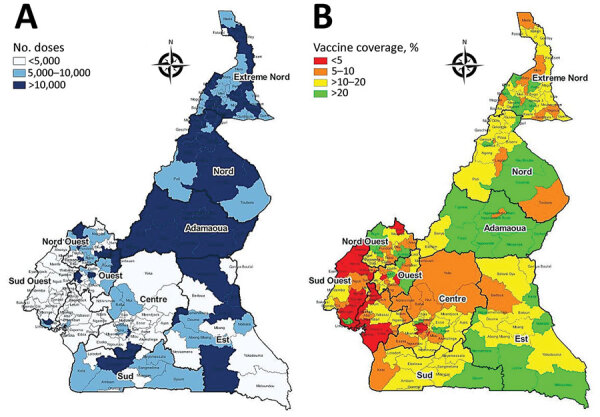
COVID-19 vaccination coverage, by health district, Cameroon, July 2022. A) Number of vaccine doses administered; B) percentage of population that has received >1 vaccine dose. Source: Cameroon Ministry of Health/Expanded Programme on Immunization.

After the emergency authorization of COVID-19 vaccines (*19*), Cameroon began preparations for vaccine introduction and implementation as part of its response strategy. Surveys on vaccine acceptance indicated most persons in Cameroon were reluctant to receive a COVID-19 vaccine even if proven to be safe and efficacious (*20*,*21*). CDC supported developing national guidelines for vaccine rollout, a nationwide vaccine deployment plan, and training manuals and communication tools to increase vaccine uptake. Since the introduction of COVID-19 vaccines in Cameroon during April 2021, the MOH has administered vaccines at fixed vaccination sites, conducted mass vaccination campaigns across all 10 regions of the country, and introduced mobile vaccination teams to increase accessibility. However, vaccine coverage remained suboptimal (Figure 3) and substantially lower than global coverage targets (*7*,*22*). Knowledge, attitudes, and practices information obtained by CDC and the MOH during October‒December 2021 showed high levels of distrust and limited knowledge about the safety and efficacy of COVID-19 vaccines. To address this issue, CDC provided surge support and additional funding for COVID-19 vaccine implementation as part of Global VAX (*23*). Efforts are ongoing to address vaccine hesitancy and increase access to vaccination services, including setting up more vaccination posts, training additional vaccinators and community health workers, and developing strategies to reach priority and hard-to-reach populations.

Surveillance and response for adverse events following immunization are essential for ensuring the safety of vaccines (*24*). With funding and technical support from CDC, the Expanded Program on Immunization in Cameroon is conducting a cohort monitoring study on COVID-19 vaccine adverse events following immunization, which will inform and strengthen vaccination efforts. A major lesson learned was the need for early engagement of community leaders, social groups and faith-based organizations to promote vaccine uptake and to widely disseminate information in local languages, adapted for each target audience.

Epidemiologic data are needed to understand the magnitude and effect of the pandemic, predict future trends, and ensure an effective public health response. Because of limited diagnostic testing during earlier waves of the COVID-19 pandemic and the large number of persons who had asymptomatic or mildly symptomatic infections, the reported number of cases globally is much lower than the actual prevalence (*25*). In collaboration with the Cameroon MOH, CDC conducted a nationwide survey to determine the seroprevalence of SARS-CoV-2 in Cameroon, identify risk factors for infection, and assess knowledge and attitudes about COVID-19 (*26*). The serosurvey was conducted during October‒December 2020 across all 10 regions of Cameroon and showed an overall estimated seroprevalence of 10.5% and regional variation ranging from 7.7% to 12.6%. The results of that survey have informed program planning and guided decision making in Cameroon.

**Figure 4:**
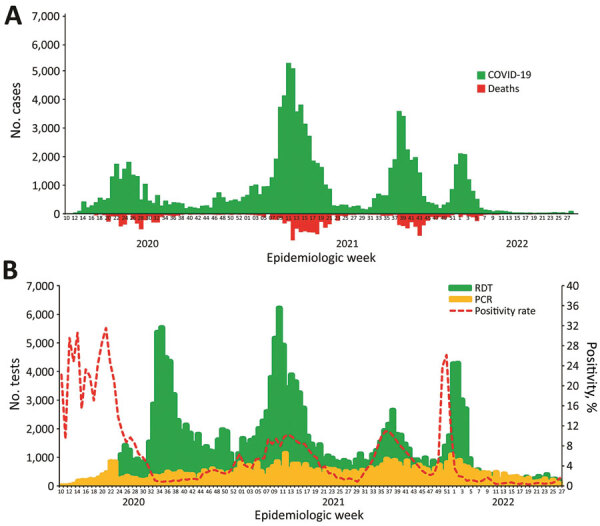
COVID-19 epidemic curve (A) and B) SARS-CoV-2 test positivity (B), Cameroon, through July 2022.Source: Cameroon Ministry of Health/Public Health Emergency Operations Center. RDT, rapid diagnostic test.

**Figure 5:**
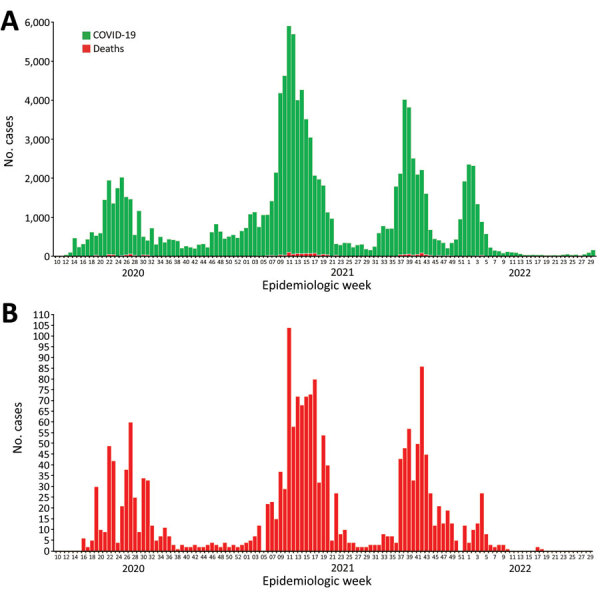
COVID-19 cases (A) and deaths (B), Cameroon, through July 2022.

As of July 2, 2022, Cameroon had recorded 120,068 cumulative confirmed COVID-19 cases, including 1,931 deaths (case-fatality rate 1.6%) (*2*). The country has had 4 waves of the epidemic, and surges in cases have been attributable to limited compliance with community mitigation measures, increased congregation and travel during holidays and festive periods, resumption of schools, and circulation of more easily transmissible variants of the virus. After Cameroon hosted the 2021 African Cup of Nations, the largest international soccer tournament on the continent, in January and February 2022, the country fully emerged from the fourth wave of the pandemic and has maintained low community transmission since (Figure 4). Rapid test and real-time PCR test positivity is <2%, community transmission remains low (Figure 5), and hospital bed availability and health staffing capacity are sufficient across all 10 regions of the country. On the basis of systems previously in place and capacity strengthened during the pandemic, Cameroon is well-positioned to respond to subsequent waves of the pandemic.

## Ensuring Health Service Provision During COVID-19 Pandemic

The COVID-19 pandemic overwhelmed health systems globally and adversely affected health programs because available resources were focused on responding to the pandemic (*3*). In Cameroon, delivery and uptake of health services were reduced because many health facilities were repurposed as COVID-19 treatment centers. To decentralize the response to all regions, the Cameroon MOH designated 78 existing health facilities at the national and subnational levels as COVID-19 isolation and treatment centers, including reference hospitals for management of critical case-patients. This change resulted in reduced delivery of primary care and other services typically provided in health facilities.

In addition, the HIV and tuberculosis programs showed decreases in testing, treatment initiation, and retention because clients were reluctant to come to health facilities because of a fear of becoming infected with SARS-CoV-2. The MOH suspended facility-led community activities to reduce transmission risk. Frontline workers and health service providers given a diagnosis of COVID-19 were put in isolation and persons who were close contacts of case-patients underwent mandatory quarantine, reducing staffing capacity and increasing workloads for other healthcare workers.

Programs developed innovative strategies to ensure continued service delivery for clients, including mobile and satellite clinics, community treatment dispensation, and home-based care to mitigate the effect of the COVID-19 pandemic on the health sector. CDC provided recommendations to the MOH to lift restrictions on community activities and enable differentiated service delivery, including HIV index case testing, antiretroviral (ART) and tuberculosis treatment dispensation, and HIV viral load sample collection in the community. Clinical implementing partners collaborated with community-based organizations and satellite health facilities for HIV testing, linkage, and treatment dispensation. CDC conducted weekly virtual clinical program and data reviews with implementing partners and health facilities and held quarterly virtual sessions to review program performance and share best practices. Establishing virtual trainings and weekly granular site management (*27*) enabled near–real-time monitoring of program activities and addressed some challenges presented by COVID-19 in delivering HIV and tuberculosis services. When feasible, the team conducted site visits, in-person data quality assessments, and partner monitoring while ensuring adequate protection to reduce the risk for COVID-19 transmission.

HIV service provision and program performance decreased in many President’s Emergency Plan for AIDS Relief‒supported sub-Saharan African countries during the COVID-19 pandemic (*28*,*29*), including decreases in pediatric and adolescent HIV testing and diagnoses. However, by scaling up differentiated service delivery models, implementing weekly monitoring of program performance, and providing virtual technical support to implementing partners and health facilities, Cameroon maintained service delivery to clients and sustained programmatic gains (*30*). Index testing performance and yields increased by 32% for pediatric HIV testing and 6% for pediatric HIV diagnoses. As a result of intensified efforts by CDC to optimize HIV program performance, the clinical program a major increase in transition of patients to receiving tenofovir, lamivudine, and dolutegravir (TLD), the recommended first-line ART regimen. Through high-level advocacy to revise national guidelines and rapidly scale-up use of TLD, the proportion of persons living with HIV (PLHIV) receiving TLD increased from 0.3% in December 2019 to 56% in December 2020 and surpassed the 80% national target by June 2021. The rapid TLD transition led to a major improvement in viral load suppression to 93% nationally. CDC provided expert technical support to Cameroon MOH and partners to scale up effective strategies across the clinical cascade, supporting HIV testing for 1,559,727 persons, identifying 62,340 HIV positive cases, and initiating treatment for 58,122 PLHIV in 2021. As of January 2022, there were 390,100 PLHIV in Cameroon receiving ART. The ART program had attained a clinical cascade achievement of 85–93–93 based on programmatic data, putting Cameroon on track to reach the 95–95–95 targets (95% of PLHIV are aware of their status, 95% of diagnosed PLHIV are receiving ART, and 95% of PLHIV receiving treatment are virally suppressed) before the 2030 timeline and achieve sustained HIV epidemic control (*31*).

## Conclusions

The COVID-19 pandemic called attention to health system gaps and underscored the need for resilient health systems to effectively respond to health threats while ensuring continued health service delivery. The effect of the pandemic has been serious in central Africa and other resource-limited settings, largely caused by the limited health infrastructure and capacity in the region. Building on the strong partnership with the Cameroon MOH, capacity established through global health programs of CDC was leveraged to support the COVID-19 response in Cameroon while implementing innovative strategies such as differentiated service delivery and granular site management to mitigate the effect of the pandemic on health programs. In addition to the need for strengthened disease preparedness and response capacity, key lessons learned from the response in Cameroon to the pandemic include the need for a well-trained and fit-for-purpose health workforce, timely mobilization of resources, and the need for coordination of multiple stakeholders to effectively manage response efforts.

Despite ongoing security and humanitarian crises in Cameroon, efforts of CDC have helped to strengthen the health system and improve health outcomes by ensuring continuity of HIV and tuberculosis services during the COVID-19 pandemic and maintaining programmatic gains. Through support of CDC, Cameroon has accelerated progress to reach the 95–95–95 targets and is positioned to be the first country in West and Central Africa to achieve HIV epidemic control. As part of the mission of the US Government to improve health globally, CDC continues to provide support to Cameroon to respond to the COVID-19 pandemic and prevent and control other public health threats. The lessons learned from Cameroon might be applicable to other resource-limited settings and conflict-affected areas to respond to COVID-19 and prepare for future pandemics.

Members of the Centers for Disease Control and Prevention Cameroon Team: Leonard C. Keleko, Ebako Takem, Christopher Coox, Adebowale Okunrinboye, Fabrice D. Nembot, Edwin Sah, Mohamadou Awalou, Yvan D. Mouzong, Anula C. Acho, Alexandre Forbin, Jeannette E. Bessem, Sidouanne Signing, Marie G. Dima, Peter T. Atonkah, Yaya N. Sale, Eric Kamleu, Richard Olemba, Johnson Teboh, Chrysantus Egbe, and Keanu Renee-Glover.
